# *SHORT INTERNODE (SHI)-Related Sequence* Genes in Bread Wheat: Molecular Characterization and Expression Analyses Suggest Their Role in Abiotic Stress Response

**DOI:** 10.3390/ijms27073269

**Published:** 2026-04-03

**Authors:** Shivanand Suresh Dudhagi, Garima Pathak, Yashraaj Sharma, Praveen Chandra Verma, Jagtar Singh, Santosh Kumar Upadhyay

**Affiliations:** 1 Department of Botany, Panjab University, Chandigarh 160014, India; shivadudhagi03@gmail.com (S.S.D.); yashraajsharma1@gmail.com (Y.S.); 2 Department of Biotechnology, Panjab University, Chandigarh 160014, India; 3 Department of Botany, B. D. College, Patliputra University, Patna 800001, India; garimapathak1309@gmail.com; 4 CSIR-National Botanical Research Institute, Rana Pratap Marg, Lucknow 226001, India; 5 Academy of Scientific and Innovative Research (AcSIR), Ghaziabad 201002, India

**Keywords:** bread wheat, abiotic stress, SHI/STY family, SRS, transcription factors, drought, salinity

## Abstract

SHORT INTERNODE (SHI)-related sequence (SRS) transcription factors are plant-specific zinc-finger proteins increasingly implicated in growth and abiotic stress responses. Despite their diverse vital role in plants, they are largely unexplored in bread wheat. In this study, we identified 15 *TaSRS* genes and classified them into five homoeologous groups in the bread wheat genome. Each TaSRS protein consisted of conserved RING-like zinc-finger and IGGH domains. The synteny and phylogenetic analyses provided insight into the evolutionary divergence and conservation of TaSRS proteins. Promoter analysis revealed the presence of stress-responsive *cis*-regulatory elements along with various transcription factor binding sites, indicating their plausible roles in drought and salinity stress responses and signalling. Additionally, the predicted regulation of a few *TaSRS* genes through certain miRNAs involved in hormone and stress responses, plant development, and nutrient uptake suggested their diverse functions. In silico protein–protein interaction and gene ontology analyses further anticipated an association of TaSRS proteins with organ development and hormone and stress response. High-throughput transcriptomic profiling revealed differential expression of *TaSRS* genes across various vegetative and reproductive stages and abiotic stress conditions. The qRT-PCR analyses confirmed the stress-responsive role of *TaSRS1-1D*, *TaSRS2-3D*, *TaSRS4-7A*, and *TaSRS5-7A* under drought and salinity conditions. These results indicated the potential role of *TaSRS* genes in stress adaptation and opened up opportunities for their detailed functional characterization and applications in the development of salinity and drought resilience in crops.

## 1. Introduction

Bread wheat (*Triticum aestivum* L.) ranks among the most significant staple crops worldwide, providing a primary source of nutrition for a substantial proportion of the population. The crop’s prevalence compared to others can be attributed to its cost-effectiveness and substantial nutritional benefits. Wheat productivity is significantly susceptible to environmental challenges, especially abiotic stresses. Salinity stress, primarily due to elevated sodium chloride (NaCl) levels, induces osmotic stress, disrupts ion equilibrium, and initiates oxidative damage. These factors collectively lead to inhibited growth, leaf chlorosis, early senescence, and significant reductions in yield [1]. Drought stress is a significant limiting factor in arid and semi-arid regions, where water scarcity constrains photosynthesis, affects plant growth, and induces oxidative stress, thereby reducing yields. The severity of the situation increases when drought and salinity co-occur, as these stresses frequently intersect in their physiological and molecular pathways, exacerbating the damage to wheat plants [2].

Wheat plants have developed complex molecular, physiological, and biochemical mechanisms to endure these stresses, which are mainly regulated by transcription factors (TFs). TFs interact with specific *cis*-regulatory elements of target gene promoters and regulate their expression. TFs regulate various adaptive responses, including osmotic adjustment, ion transport, antioxidant defence, and hormone signalling [2,3,4]. Various TF families have been identified as crucial in salinity stress response. DREB/CBF proteins regulate ABA-independent stress pathways, while NAC and WRKY transcription factors modulate ROS detoxification and ion homeostasis. Additionally, the bZIP and MYB transcription factors primarily function in ABA-dependent signalling to regulate osmolyte biosynthesis and stress-protective proteins [4,5]. The findings underscore the pivotal function of transcription factors as primary regulators of intricate gene networks that facilitate wheat’s tolerance to salinity stress.

Among the various TF families, the SHORT INTERNODES (SHI)-related sequence (SRS) family, also known as the SHI/STY family, has recently attracted attention for its roles in regulating growth and stress responses. The SRSs represent a plant-specific group of transcription factors consisting of two highly conserved domains: an N-terminal RING-like zinc-finger domain and a C-terminal IGGH domain [6,7]. The RING-like zinc-finger domain, also known as the RING domain, comprises the “C-X2-C-X7-C-X4-C-X2-C-X7-C” motif. The RING domain is engaged in numerous physiological and biochemical processes in plant cells. The C-terminal IGGH domain is rich in acidic amino acid residues and is supposed to facilitate protein dimerization. Interestingly, the IGGH domain appears unique to the SRS family, suggesting specialized regulatory roles [6,8,9].

The SRS family genes have been identified in numerous plant species, including Arabidopsis, maize, rice and others [6,7,10,11,12]. In Arabidopsis, this family comprises 10 members, including LRP1 (Lateral Root Primordium 1), SHI, and STY1 (STYLISH1), which exhibit functional redundancy. All of them consist of both the RING-like zinc-finger and IGGH domains, except SRS8 that lacks the IGGH domain [6,13,14]. AtSTY1 (SRS1) is crucial for the development of apical meristematic tissue due to its involvement in the biosynthesis of auxin [8]. AtLRP1 regulates root primordium formation via auxin modulation [15,16]. AtSHI influences gibberellin signalling and floral development processes, whereas AtSRS5 is responsible for regulating photomorphogenesis and inhibiting lateral root formation [6,14,17].

In monocots such as maize, rice, and barley, the *SRS* genes participate in developmental regulation; they influence awn elongation and pistil morphology, inflorescence architecture, and carbohydrate redistribution during senescence [10,18]. The ZmRUM1 protein, a part of the Aux/IAA-ARF unit, has the capacity to directly interact with the *ZmLRP1* promoter, thereby suppressing its expression [19]. In rice, *SHI1* regulates plant architecture by modulating the transcription factor *OsIPA1* that governs tiller and panicle development [20].

Beyond development, increasing evidence suggests that the *SRS* genes also regulate abiotic stress responses, including salinity and drought. In rice, SHI1 coordinates growth stress trade-offs by integrating auxin, abscisic acid (ABA), and brassinosteroid (BR) signalling [20]. In soybean, GmSRS18 negatively regulates drought and salt tolerance [21]. In alfalfa, numerous *MsSRS* genes were strongly induced by cold and salt, indicating tissue-dependent stress regulation [22]. The *MaSRS* genes of sweet clover exhibited significant modulation when exposed to methyl jasmonate (MeJA), salicylic acid (SA), low temperature, and salinity [23]. In cotton, GhSRS21 negatively influences salt tolerance by operating through a mechanism that relies on reactive oxygen species metabolism [24].

Despite the diverse vital roles of SRS TFs in plants, they are still largely unexamined in bread wheat. In an earlier study, Yu et al. [25] identified TaSRS genes, but the study lacks proper nomenclature in accordance with international guidelines for wheat gene nomenclature and provides limited characterization of the genes, including their regulatory mechanisms. Hence, we carried out the comprehensive characterization of *TaSRS* genes across the A, B, and D sub-genomes of allohexaploid bread wheat (*Triticum aestivum* L.). The identified TaSRSs were analyzed for various key features, such as gene structure, conserved domains and motifs architecture, physicochemical properties, and evolutionary relationships. Additionally, we examined the occurrence of *cis*-regulatory elements (CREs), transcription factor binding sites (TFBSs), predicted miRNA interactions, protein–protein interaction (PPI) networks, and gene ontology (GO) enrichment to gain insight into their functions. Furthermore, the association of *TaSRS* genes with developmental processes as well as abiotic stress responses was examined using available high-throughput transcriptomic data. The roles of a few *TaSRS* genes under drought and salinity stress were also examined using qRT-PCR. This study provides detailed information on *TaSRS* genes in bread wheat, which will facilitate the functional characterization of each gene in future studies.

## 2. Results and Discussion

### 2.1. Identification, Chromosomal Distribution and Structure Analysis of TaSRS Genes

**Figure 1:**
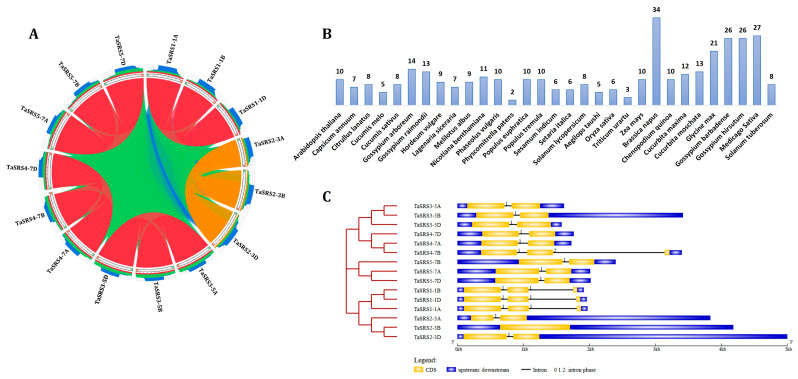
**Homoeologous grouping and gene structure organization in *TaSRS* genes.** (**A**) The figure shows homoeologous grouping among *TaSRS* genes located on A, B, and D sub-genomes of bread wheat. Syntenic relationship was analyzed using the Circoletto web tool (25.03.23). (**B**) Histogram illustrating the number of *SRS* genes identified in various plant species. (**C**) The figure shows exon, intron, and UTR organization of *TaSRS* genes, constructed using the Gene Structure Display Server 2.0. Exons, introns and UTRs are shown by a yellow box, a black line and a blue box, respectively. The numbers 1 and 2 represent the intron phases.

Gene mining is fundamental in plant genomics, as it enables the systematic identification of gene families with key physiological traits, including stress adaptation and development [26]. This is particularly important in complex genomes, like the allohexaploid genome of bread wheat, which is made up of three sub-genomes (AABBDD) [27,28]. In this study, we employed a comprehensive approach and integrated multiple bioinformatics tools to mine *TaSRS* genes. In total, 15 *TaSRS* genes were identified in the Ensemble plant database (Figure 1A, Table 1). All the identified TaSRS proteins consisted of a conserved RING-like zinc-finger domain (DUF702) (Pfam ID: PF05142), which is a characteristic feature of SRS proteins [6]. The A- and D-genome progenitors of bread wheat, i.e., *Ae. tauschii*, and *T. urartu*, comprised five and three *SRS* genes, respectively. Moreover, the number of genes identified in *Ae. tauschii* is similar to the number of genes present on the D sub-genome of bread wheat. However, the number of *TaSRS* genes on the A sub-genome is higher than that of *T. urartu*, which could be due to post-hybridisation duplication events [28]. The number of *SRS* genes varies across diploid and polyploid plant species. For instance, the diploid genome of Arabidopsis, tomato and rice consists of ten, eight and six *SRS* genes, respectively [6,7,8,11,14,29]; however, tetraploid genomes of barley and cotton comprise 34 and 26 *SRS* genes, respectively (Figure 1B) [9,24]. These results suggest the uneven distribution of *SRS* genes in various plant genomes, which could be due to variable duplication events during evolution [28].

The identified *TaSRS* genes were named using the standard guidelines (Table 1) [30]. The *TaSRS* genes formed five homoeologous groups (HGs) (*TaSRS1–TaSRS5*) based on their sub-genomic location and high sequence similarity (>97%) (Figure 1A). The *TaSRS* genes exhibit distinct patterns of conservation across the homoeologous groups. These genes are mapped on the four different chromosomes (1, 3, 5, and 7) across the A, B, and D sub-genomes. Each sub-genome contributed an equal composition of *TaSRS* genes. Chromosome 7 consists of two homoeologous group genes (*TaSRS4* and *TaSRS5*), while *TaSRS1*, *TaSRS2* and *TaSRS3* homoeologous group genes were present on chromosome 1, chromosome 3, and chromosome 5, respectively (Figure S1). Similarly, the *TaSRS* genes are distributed across different chromosomes in various plants, including rice, maize, and others [10,11,12,29].

The gene structure analysis reveals various characteristic features, including the occurrence of UTRs, the number of exons, the pattern and phases of introns, and the distribution of *cis*-regulatory elements in promoter regions. In addition, it also provides evolutionary relatedness among the various genes of the gene family.

The majority of *TaSRS* genes consisted of two exons interrupted by one intron of variable length; however, *TaSRS1* homoeologous group genes and *TaSRS4-7D* consisted of three exons and two introns, in which intron 1 is similar to the other genes in length (Figure 1C). Variation in exon-intron structure may arise from exonization or pseudoexonization, insertions or deletions, and gains or losses, and these changes play a critical role in the functional diversification of genes [31]. All the introns were in phase 1, except for a phase 2 intron in *TaSRS4-7B*. Earlier studies have shown that the phase 1 introns are disproportionately enriched in plant stress-related genes, ensuring splicing fidelity and enabling regulatory flexibility. In the *TaSRS* gene family, the dominance of phase 1 introns suggests a conserved ancestral origin and intense selective pressure, likely reflecting their role in precise splicing and stress-responsive regulation [32]. This indicates structural conservation, with phase 1 introns being the most prevalent, suggesting stability that minimizes disruptions during exon shuffling [33]. Except for *TaSRS1*, the homoeologs of genes belonging to *TaSRS2*, *TaSRS3*, *TaSRS4*, and *TaSRS5* exhibit a compact architecture comprising two closely spaced exons and relatively short intronic regions (Figure 1C). This compact structure in the gene is recognized for helping rapid and efficient gene expression, a trait often found in plant transcription factors associated with stress responses, as evident in the studies of *SRS* genes in cotton and rapeseed [9,24].

### 2.2. Basic Physicochemical Properties of TaSRS Proteins

Physicochemical properties of proteins play an important role in determining their structure, stability and biological function. Parameters such as molecular weight, isoelectric point, hydrophobicity and aliphatic index provide crucial insights into protein behaviour under different physiological conditions. Understanding these properties helps in predicting protein solubility, localization, and potential interactions, supporting both structural and functional characterization.

A rigorous in silico investigation using the Expasy-Protparam tool revealed numerous physicochemical properties of TaSRS proteins (Table 1, Table S1). The length of TaSRS proteins ranged between 247 and 357 amino acid (AA) residues. TaSRS2-3A (247 AA) was the smallest, while TaSRS2-3B (357 AA) was the longest protein. Their MW ranged between 25.09 and 35.48 kDa, which is consistent with SRS proteins reported in various plant species, including Arabidopsis, rice, tomato, cotton, and soybean [6,7,11,14,21,29]. The pI values ranged from 7.70 to 8.96, indicating a distinctly basic nature for most TaSRS proteins. This basic nature is crucial, as it ensures a net positive charge at nuclear pH, thereby facilitating strong electrostatic binding to negatively charged DNA [34]. Further, all the TaSRS proteins contained an NLS (R/KRRER/K) and were predicted to be nuclear localized. Similar properties of SRS proteins, such as their basic nature and NLS, have also been reported in other plant species, including rice and soybean [11,21].

The negative GRAVY values (−0.684 to −0.276) indicated the hydrophilic nature of TaSRS proteins (Table S1) and support their solubility within the aqueous nuclear environment [35]. An instability index of more than 40, along with low-to-moderate aliphatic index values (38.86–56.36), indicated the unstable nature of TaSRS proteins. Such instability and structural plasticity are characteristics of the regulatory proteins, which facilitate rapid synthesis and proteasomal degradation. This dynamic turnover plays a crucial role in transcriptional reprogramming during stress adaptation [36].

### 2.3. Domains and Motifs Analyses

**Figure 2:**
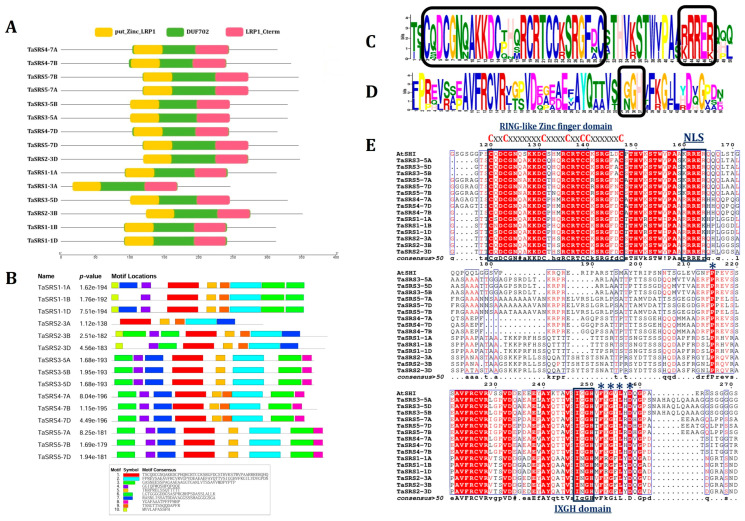
**Domain architecture, conserved motif and MSA analyses of TaSRS proteins.** (**A**) The figure illustrates the domain architecture and distribution in TaSRS proteins. Data obtained from NCBI-CD search and visualized using TBtools. (**B**) The figure shows the distribution of conserved motifs in TaSRS proteins identified by the MEME Suite. The consensus sequences for each motif are listed and shown in different colours. (**C**) The sequence logo represents the conserved RING-like zinc-finger domain and NLS. (**D**) The sequence logo shows a conserved IXGH domain. (**E**) The figure shows a multiple sequence alignment of SRS proteins from *T. aestivum* and AtSHI (as a representative), showing a conserved RING-like zinc-finger domain, a monopartite NLS, an IXGH domain, and several conserved amino acid residues (indicated by *). MSA analysis was carried out using the Multalin tool.

The domain architecture of TaSRS proteins was conserved. All of them consisted of three domains: DUF702 (PF05142), N-terminal putative zinc-finger (RING-like zinc-finger) (put_zinc_LRP1; IPR006510) and a C-terminal LRP1 domain (LRP1_Cterm; IPR006511). The N-terminal putative zinc-finger and C-terminal LRP1 domains overlap the DUF702 (Figure 2A, Table S2). A similar architecture of SRS proteins has also been reported in other plant species, such as Arabidopsis and rice [6,7,11].

Additionally, we performed conserved motif analysis using MEME2.0 and multiple sequence alignment (Figure 2B–E). Motifs one and two were conserved in all the TaSRS proteins, which represent the RING-like zinc-finger, including the nuclear localisation signal (NLS), and IXGH domain, respectively. TaSRS1-1B lacks motif 7; however, it is located at the C-terminal region of TaSRS2 homoeologous proteins. Motif 10 was present only in homoeologs of TaSRS1 and TaSRS2, and motif 8 was specific to homoeologs of TaSRS3, TaSRS4 and TaSRS5.

The MSA revealed the conserved nature of TaSRS proteins, including conserved AA residues and structural features (Figure 2E). The RING and IXGH domains were found to be highly conserved, reaffirming the necessity for DNA binding and transcriptional activity [6,7,8,14]. The monopartite NLS (R/KRRER/K) was found to be conserved in all TaSRS sequences, suggesting a conserved mechanism for their nuclear translocation [37]. Additionally, P (215), F (252), G (254), L (256), and D (258) AA residues were also found conserved in all the TaSRS proteins; however, their functions need to be explored in future studies.

### 2.4. Evolutionary Analyses of TaSRSs

**Figure 3:**
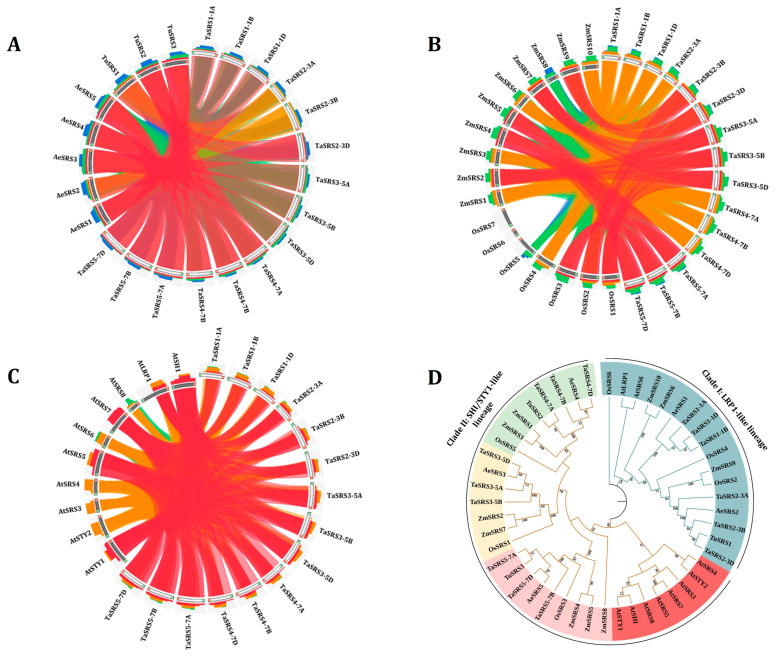
**Syntenic and phylogenetic relationship between SRS proteins of *Triticum aestivum* and other plants.** The figure shows orthologous or syntenic pairing of *SRS* genes in *Triticum aestivum* with (**A**) *Ae. tauschii* and *T. urartu* (AeSRS, TuSRS), (**B**) *O. sativa* and *Z. mays* (OsSRS, ZmSRS), and (**C**) *A. thaliana* (AtSRS). Circos-style syntenic maps were generated using the Circoletto web tool. Coloured ribbons indicate orthologous gene pairs. (**D**) The figure illustrates the phylogenetic relationship among the SRS proteins in *Ae. tauschii*, *A. thaliana*, *O. sativa*, *T. aestivum*, *T. urartu* and *Z. mays.* The phylogenetic tree shows two major clades, further divided into five groups, each shown in a different colour. The phylogenetic tree was constructed in MEGA11 using the neighbour-joining method and 1000 bootstrap replicates. >50% bootstrap support values shown at the corresponding nodes, where higher values reflect more reliability.

To explore evolutionary relatedness, the syntenic and phylogenetic relationships of the TaSRS proteins were examined with those of the SRS proteins from *Ae. tauschii*, *T. urartu*, *A. thaliana*, *O. sativa*, and *Z. mays* (Figure 3). The synteny with the wild ancestors of D and A sub-genomes of bread wheat, i.e., *Ae. tauschii* and *T. urartu*, respectively, revealed strong homology among the related SRS proteins, indicating well-known shared ancestry. For instance, the TaSRS2 homoeologous proteins showed a close relationship with the AeSRS2 and TuSRS1 (Figure 3A).

The synteny analyses with rice and maize SRS proteins revealed a conserved orthologous relationship. We found that the TaSRS1 and TaSRS2 homoeologous group proteins were orthologous to OsSRS2 and ZmSRS9, while the TaSRS3 homoeologous proteins corresponded to OsSRS1, ZmSRS2 and ZmSRS7. The TaSRS4 homoeologous proteins shared orthology with ZmSRS3, ZmSRS2, and OsSRS1, while TaSRS5 shared orthology with OsSRS3, ZmSRS4, and ZmSRS5. Surprisingly, OsSRS6 formed a separate branch, suggesting species-specific divergence in rice (Figure 3B). The cross-family synteny analysis with Arabidopsis revealed that the homoeologous groups of TaSRS1 and TaSRS2 aligned with AtLRP1, while homoeologs of TaSRS3, TaSRS4 and TaSRS5 shared an orthologous relationship with AtSHI, AtSTY1, and AtSRS5 (Figure 3C). Similar syntenic relationship among the genes of bread wheat, rice, maize, and Arabidopsis was also reported in earlier studies [38,39].

The phylogenetic tree was constructed using full-length SRS sequences from various plant species and revealed a clear evolutionary relationship across the species (Figure 3D). The neighbour-joining analysis revealed two major evolutionary clades: Clade I-LRP1-like and Clade II-SHI/STY-like, corresponding to the functional specialization established in the Arabidopsis SRS family proteins. The role of AtLRP1 in regulating lateral root growth, auxin homoeostasis and photomorphogenesis has previously been demonstrated [15,16]. Clustering of *TaSRS1* and *TaSRS2* HG genes, ZmSRS6, ZmSRS10, OsSRS4 and OsSRS9 with AtLRP1 indicates their role in similar processes. AtSHI, AtSTY1, and AtSTY2 genes are known to regulate floral development, carpel formation, and auxin biosynthesis [7,40,41]. This clade includes several monocot SRS proteins, such as *TaSRS3*, *TaSRS4* and *TaSRS5* homoeologous group genes, that are likely to retain their regulatory roles in the regulation of reproduction and biosynthesis of auxin. Furthermore, the homoeologous *TaSRS* genes showed tight clustering among them and with the orthologous genes from *Ae. tauschii* and *T. urartu*, which could be due to an ancestral relationship among them. In addition, some of the *TaSRS* genes, such as *TaSRS2* and *TaSRS3* homoeologous groups, were closely clustered with OsSRS2 and ZmSRS9, indicating conserved monocot functions. Furthermore, the TaSRS homoeologs were distributed across multiple branches, suggesting their subfunctionalization.

### 2.5. Promoter Analyses

**Figure 4:**
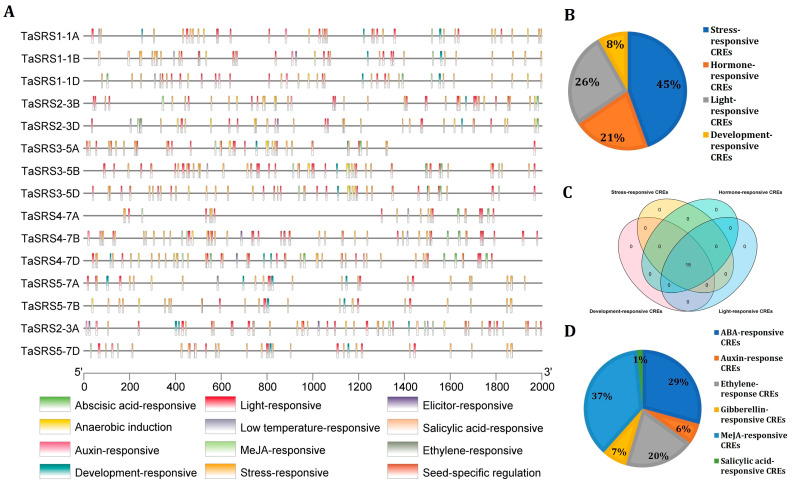
**In silico *cis*-regulatory element analysis in the promoter region of *TaSRS* genes.** (**A**) The figure shows the distribution of putative *cis*-regulatory elements in the 2000 bp upstream promoter regions of *TaSRS* genes. The CREs were grouped based on their biological function, and each group is represented in a different colour, as indicated in the legend below. (**B**) The pie chart shows the relative proportion of stress, hormone, light and development-responsive CREs in *TaSRS* promoters. (**C**) The Venn diagram illustrates the distribution of four functional categories of CREs in *TaSRS* promoters. All 15 *TaSRS* genes contain CREs belonging to each functional category. (**D**) The pie chart shows the relative proportion of different hormone-responsive CREs in the upstream region of *TaSRS* genes. The putative CREs were identified using the PlantCARE database. The distribution landscape and the Venn diagram were generated using TBtools software.

To understand the transcriptional regulation of TaSRS genes, the occurrence of cis-regulatory elements (CREs) and transcription factor binding sites (TFBS) was analyzed. *TaSRS* promoter sequences contained a wide range of CREs (Figure 4A, Table S3), which could be broadly divided into four categories. The majority of them were stress-responsive (45%), followed by hormones (26%), light (21%), and growth and development-related (8%), and were found across all *TaSRS*s (Figure 4B,C).

The stress-responsive CREs included the components of the ABA signalling pathway (ABRE, ABRE3a, and ABRE4), which is a central mediator of plant responses to drought and salinity stress (Table S3) [4,42]. Similarly, DRE core and DRE1 elements known to regulate dehydration and salt-inducible gene expression were present in almost all the TaSRS promoter sequences, suggesting ABA-independent activation of SRS genes during drought and salt stress in wheat [43]. The pronounced presence of MYB and MYC binding sites further supports the role of TaSRS genes under osmotic and oxidative stress conditions. Other stress-responsive CREs include the WUN motif, involved in the wounding response and low-temperature response (LTR), the drought-related MYB site (MBS), and TC-rich repeats, which are widely associated with general defence mechanisms (Table S3). The presence of TCA elements, ERE, TGACG motif, and CGTCA motif suggest the association of TaSRS gene with pathogen response and systemic-acquired resistance [4,44,45,46].

Consistent with previous reports from Brassica and tomato, the *TaSRS* promoters contain motifs for meristematic activity (CAT-Box) and seed-specific CREs (O2 site and RY element). The light-responsive elements, such as the GATA motif, Sp1, LAMP element, and G-box element, distributed across the *TaSRS* gene family, suggest their possible role in responding to light fluctuations and photomorphogenic regulation [4,9,29], underpinning the role of TaSRS transcription factors in photosynthesis and developmental programmes. Among the hormone-responsive CREs, methyl jasmonate (37%) and ABA-responsive CREs (29%) constituted one third of the CREs (Figure 4D), suggesting a fine-tuned cross-talk between abscisic acid, jasmonic acid, salicylic acid, and auxin pathways. Earlier studies have demonstrated that the ABRE- and jasmonate-responsive motifs confer stress-inducible regulation in SRS gene expression during drought, salinity, and biotic stress conditions [4,43,46].

**Figure 5:**
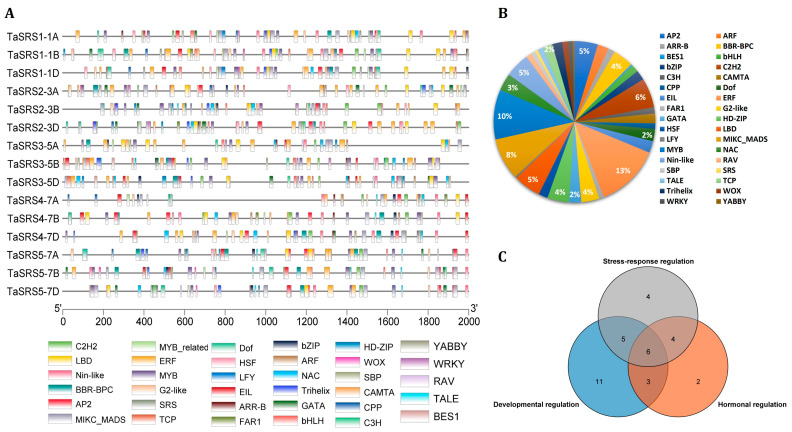
**Predicted Transcription factor binding sites in the promoter of *TaSRS* genes.** (**A**) The figure shows the distribution of predicted TFBSs in the 2000 bp upstream promoter region of *TaSRS* genes. Each transcription factor is represented by a different colour box, as indicated in the legend below. (**B**) The pie chart shows the relative proportion of these TFBSs predicted in the *TaSRSs* promoter. (**C**) The Venn diagram shows overlap among functional categories of predicted TFBSs in *TaSRS* promoters. TFBSs were predicted using the PlantRegMap web tool. The distribution patterns of predicted TFBSs and the Venn diagram were generated using TBtools software.

Comprehensive mapping of TFBS identified 810 predicted sites across 35 TF groups in the *TaSRS* promoter sequences, revealing pronounced diversity and gene-specific regulatory complexity (Figure 5A, Table S4). The occupation of TFBS was dominated by TFs, ERF (12.8%), MYB (10%), MIKC_MADS (8%), C2H2 (6%), and AP2, NIN-like and LBD (5%). Additionally, various stress-responsive TFBS, such as NAC, bZIP, EIL, HSF, WRKY, etc., were also identified in the majority of *TaSRS* promoter sequences (Figure 5B). Of the total identified TF groups, 19 belong to stress response, 15 to hormonal regulation, and 25 to developmental regulation (Figure 5C). ARF, BES1, ERF, and AP2 TFs are known for their roles in auxin, brassinosteroid, ethylene, and ABA signalling, respectively. Various TFs, such as CAMTA, MYB, NAC, and WRKY, play crucial roles in abiotic stress responses, including drought and salinity stress [2,4,5]. These findings suggest the association of various *TaSRS* genes with abiotic stress signalling. The presence of developmental regulators such as MIKC_MADS, C2H2, RAV, and TALE suggests the involvement of *TaSRS* genes in developmental pathways. The presence of multifunctional regulators such as MYB, AP2/ERF and NAC in all the *TaSRS* promoters suggest coordinated regulation of these genes across diverse adaptation and developmental processes [44,45].

### 2.6. miRNA Interactions

**Figure 6:**
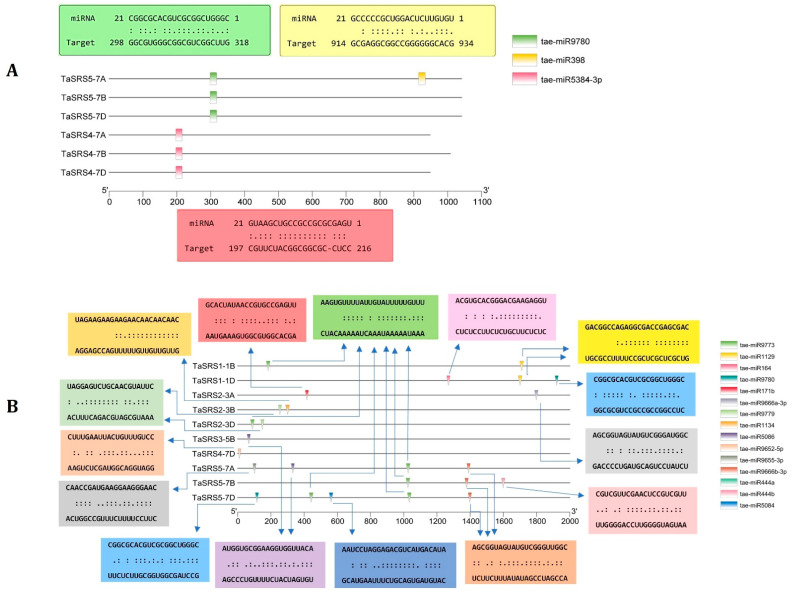
**Predicted miRNA-mediated regulation of *TaSRS* genes.** (**A**) The figure shows the positions of predicted miRNAs binding within the CDS of *TaSRS* genes. (**B**) The figure shows the positions of predicted miRNAs within the 2000 bp upstream promoter region of *TaSRS* genes. Arrows represent the target sites of the miRNAs, and coloured boxes represent the complementary base pairing. miRNAs were predicted using the psRNATarget server and mapped using TBtools.

The miRNAs are known for post-transcriptional regulation of gene expression by complementary binding to the mRNAs in the cytoplasm. A few recent studies have demonstrated nuclear translocation of some miRNAs, which could interact with the promoter elements and modulate transcription [47]. Therefore, in the current study, we predicted the interaction of known miRNAs of bread wheat with both cDNA and promoter sequences of *TaSRS* genes (Figure 6). The in silico miRNA interaction study suggested the post-transcriptional regulation of the *TaSRS4* and *TaSRS5* homoeologous group genes by three known miRNAs of bread wheat (tae-miR398, tae-miR5384-3p and tae-miR9780) (Figure 6A). tae-miR3584-3p and tae-miR9780 showed interaction with *TaSRS4* and *TaSRS5* homoeologous group genes, respectively. These miRNAs could regulate gene expression by cleaving target mRNAs. However, tae-miR398 was predicted to target the *TaSRS5-7A* gene via a translation-inhibition mechanism. tae-miR398 and tae-miR9780 are known drought and salt stress-responsive miRNAs in wheat [48,49]. tae-miR398 is significantly down-regulated following NaCl treatment in wheat. Furthermore, miR398 exhibits differential regulation under drought conditions across wheat varieties, with upregulation in wild emmer wheat but potentially altered in cultivated species [49,50]. The interaction of these miRNAs with *TaSRS* genes indicate their role in similar stress conditions.

Multiple miRNA regulators were predicted to interact with the promoter region of various *TaSRS* genes, including tae-miR9780, tae-miR171b, tae-miR5084, tae-miR5086, tae-miR44a, tae-miR44b, etc. (Figure 6B). Strikingly, not all 15 *TaSRS* genes contain miRNA-binding sites in their promoters; only those with predicted complementarity are actively regulated. The positional distribution of miRNA-binding sites within the 2000 bp promoter region revealed clustering at specific locations; few *TaSRS* genes (e.g., *TaSRS5-7D*) contain multiple miRNA interactions dispersed across the promoter, while others (e.g., *TaSRS3-5B* and *TaSRS4-7D*) show single predicted targeting.

The identification of miRNA-binding sites within *TaSRS* promoter regions extends regulatory control beyond post-transcriptional mechanisms [51]. In wheat, where dynamic responses to drought and salinity stress are critical, promoter-targeted miRNA interactions could fine-tune *SRS* gene expression at both transcriptional and post-transcriptional levels, thereby amplifying the responsiveness of the regulatory network. The integration of miRNA-promoter interaction data with cDNA-level targeting provides information about an additional layer of control and its contribution to stress resilience in the wheat *SRS* gene family [48,49,50].

### 2.7. Protein–Protein Interactions (PPI)

PPIs are fundamental to cellular processes, enabling proteins to form dynamic networks that drive signal transduction, gene regulation, metabolic pathways and stress-adaptive responses [52]. Understanding these PPIs provides valuable insights into the complex molecular framework operating in living systems. It reveals how changes in these networks can affect plant development, physiology and stress tolerance [53]. Therefore, the characterization of PPIs is essential for understanding the core biological functions as well as for advancing the biotechnological applications, allowing the identification of key molecular regulators for precise genetic improvements [52,54].

**Figure 7:**
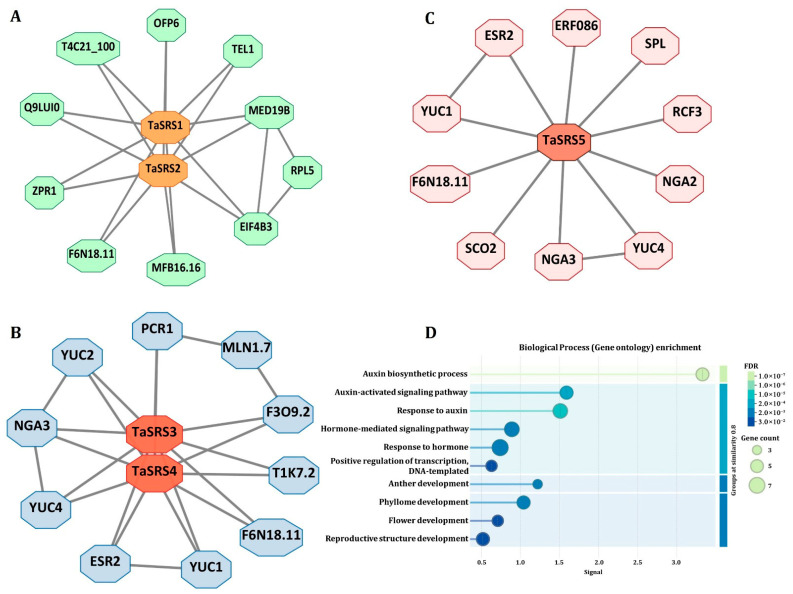
**In silico analysis of protein–protein interaction (PPI) and GO enrichment of TaSRS proteins.** The figure shows the predicted interaction networks between the identified proteins and the proteins belonging to (**A**) TaSRS1 and TaSRS2, (**B**) TaSRS3 and TaSRS4, and (**C**) TaSRS5. The homoeologous group members of each TaSRS protein were grouped, as they share a similar interaction profile. (**D**) The figure shows Biological Process (Gene ontology) enrichment of TaSRS proteins. The interacting proteins were identified using the STRING database, and the interaction network was constructed in the Cytoscape.

Previous studies in Arabidopsis, pitaya, sesame, and quinoa suggest that SRS proteins participate in diverse interaction networks that integrate hormone signalling, organogenesis, and stress response through specific protein partners. These PPI networks enable SRS transcription factors to act as molecular regulators, ensuring plant growth and adaptation to fluctuating environmental conditions [7,55,56]. Based on these foundational insights in the SRS TF family across diverse plant species, we constructed PPI networks for TaSRS proteins. Our predicted PPI analyses indicated that the TaSRSs function as central regulators in developmental, hormonal and environmental signalling networks. The homoeologous group proteins of TaSRS1 and TaSRS2 were found to interact primarily with proteins involved in cellular organization, such as MED19B, RPL5 and EIF4B3 (Figure 7A). Along with these, the homoeologs of TaSRS1 and TaSRS2 were found to interact with proteins involved in RNA processing (ZPR1) and maintenance of cellular structure (OFP6 and TEL1). These in silico interactions suggest the possible role of TaSRS1 and TaSRS2 homoeologous group proteins in coordinating the expression of genes involved in fundamental cellular processes [57,58,59,60]. The homoeologous group proteins of the TaSRS3 and TaSRS4 interaction network include the key enzymes involved in the auxin biosynthesis pathway (YUC2, YUC1, and YUC4) [61], organ development factors (NGA3 and ESR2), and proteins involved in hormonal regulation, such as PCR1 (Figure 7B) [62,63]. This suggests that TaSRS3 and TaSRS4 may participate in the formation of the reproductive structure and hormone-responsive growth in wheat.

Moreover, we found that the TaSRS5 homoeologs interact with floral regulators and proteins involved in the auxin biosynthesis pathway (SPL, YUC1, NGA2, and NGA3) [61,62,64,65]. Furthermore, they showed interactions with proteins involved in stress response (NGAs, RCF3 and ERF086) (Figure 7C) [66,67,68]. These functional diversities of TaSRS proteins indicate their role in the integration of reproductive processes with environmental adaptation. Our findings were consistent with the previous studies on SRS proteins across diverse plant species. In Arabidopsis, SHI, STY1/STY2, and LRP1 regulate organ development by modulating auxin biosynthesis and signalling via the YUCCA genes, resulting in the formation of root, gynoecium, flower, and leaf architecture [8,40,41]. In cassava, SRSs regulate auxin homeostasis during development, and respond to hormonal stimuli and abiotic stress, highlighting their roles in growth and stress adaptation [69].

Gene ontology (GO) enrichment analysis revealed that the TaSRS proteins were predominantly associated with the auxin biosynthesis and signalling pathway, along with hormone-mediated regulation and various developmental processes (Figure 7D). The most significantly enriched biological process was auxin biosynthesis, with the highest signal value (10 × 10^−7^). Similarly, the auxin-activated signalling pathway and response to the auxin were highly enriched, emphasizing the pivotal role of *SRS* genes in regulating auxin metabolism and signalling cascades. These findings were consistent with the previous reports, highlighting the participation of SRS family members in auxin-mediated regulatory networks and their central role in coordinating growth and response to abiotic stresses [12,13,21,24,29].

### 2.8. Expression Analyses of TaSRS Genes

To understand the roles of *TaSRS* genes, a comprehensive expression analysis was performed using high-throughput transcriptomic data from various vegetative and reproductive tissues, as well as under drought, heat, and salinity stress conditions. Additionally, the expression of a few selected genes under drought and salinity stress was subsequently validated using qRT-PCR with gene-specific primers (Table S5).

#### 2.8.1. Expression Analysis in Vegetative and Reproductive Tissues

**Figure 8:**
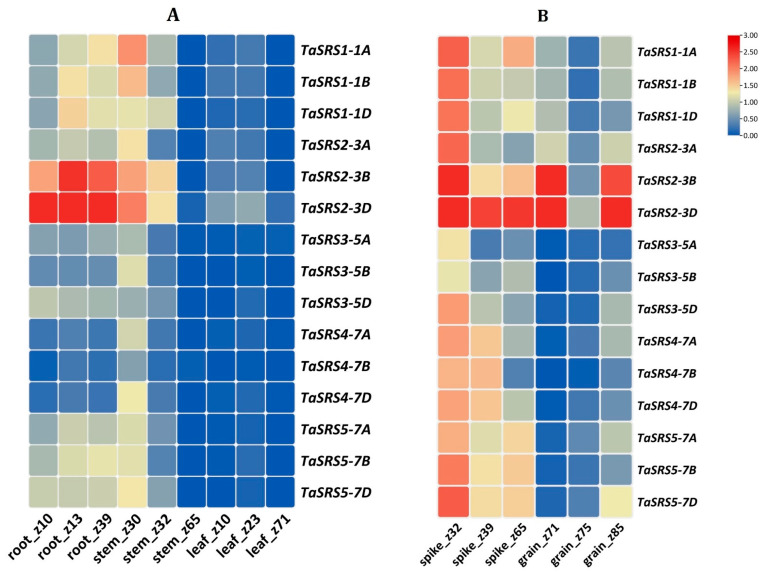
**Expression profiling of *TaSRS* genes in various vegetative and reproductive tissues.** (**A**) The figure represents the expression profiles of *TaSRS* genes in different vegetative tissues, viz., root, stem and leaves at various developmental stages. (**B**) This figure shows the expression profiles of *TaSRS* genes in reproductive tissues at multiple development stages. The fold change in expression levels was calculated against the control. The stages were categorized using the Zadoks scale. The heatmap was generated using TBtools. The high level of expression indicated by red and blue colours indicates lower expression.

In vegetative tissues, the majority of TaSRS genes were expressed across developmental stages of root and stem tissues (Figure 8A). However, their expression was significantly lower across all leaf tissue developmental stages and in the later stages of stem development. TaSRS2-3B and TaSRS2-3D were among the most abundantly expressed genes across all developmental stages of vegetative tissues. These highly expressed genes were grouped into the LRP1-like lineage (Clade I) in phylogenetic analysis, which also comprises root-associated orthologues from Arabidopsis [16]. The results suggest that this clade has retained an ancestral role in root meristem maintenance and lateral root primordia formation. The promoters of TaSRS2 homoeologous genes were enriched in auxin, gibberellin and development-related CREs, as well as MYB, NAC, bZIP, and TCP TFBSs. This provided a mechanistic explanation for their preferential expression in actively growing root tissues and integration into the auxin-centred growth regulatory network [4,16].

In the case of reproductive tissues, the majority of *TaSRS* genes showed significant expression across all spike developmental stages; however, their expression was significantly reduced during grain development, except for *TaSRS2-3B* and *TaSRS2-3D* (Figure 8B). These results indicated the role of *TaSRS* genes in the early reproductive phases, probably during pollen and ovule formation. *TaSRS2-3B* and *TaSRS2-3D* showed the highest transcript levels across the reproductive tissues, followed by *TaSRS1* homoeologous group genes. *TaSRS1* and *TaSRS2* homoeologous group genes were tightly clustered within the LRP1-like branch in the phylogenetic tree. The promoters of *TaSRS1* and *TaSRS2* HGs were found to be enriched in auxin, ABA, and general developmental CREs and to contain multiple binding sites for MADS-box, TCP, and other reproductive transcription factors, which correlate with their spike/grain expression levels. In contrast, SHI/STY-like *TaSRS3*-*TaSRS5* HG genes, despite conservation at the protein level, possess more modest or divergent CRE and TFBS landscapes and show low or unresponsive reproductive expression, suggesting sub-functionalization into specialized or partially redundant roles during floral and grain development [4,7,9,12].

#### 2.8.2. Drought and Heat-Responsive Expression

**Figure 9:**
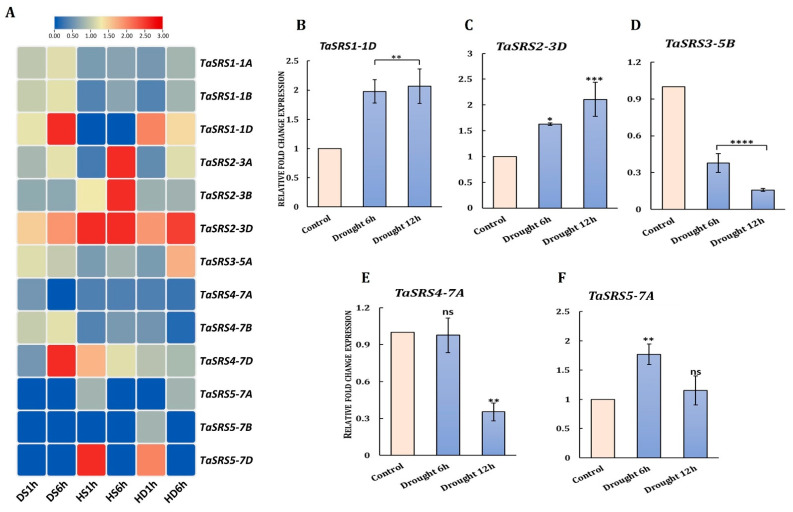
**Expression analyses of *TaSRS* genes during drought and heat stress conditions.** (**A**) The figure shows the fold change expression profiles (FPKM) of *TaSRS* genes at different time points of drought and heat stress, calculated against the control. The heatmap was generated using TBtools. The high level of expression indicated by red and blue colours indicates lower expression. (**B**–**F**) The bar graphs show transcript accumulation of selected *TaSRS* genes under drought stress at 1 and 6 h of the 20% PEG treatment. qRT-PCR was performed using total RNA of shoot tissues. *TaARF* was used as an internal control. The experiment was conducted in triplicate, and the data were represented as mean ± SD. Asterisks denote the level of significance compared with control (* *p* ≤ 0.05; ** *p* ≤ 0.01; *** *p* ≤ 0.001; **** *p* ≤ 0.0001; ns, not significant), determined by one-way ANOVA (Fisher’s and Dunnett’s post-test).

During drought and heat stress conditions, a distinct expression pattern was observed for some *TaSRS* genes (Figure 9A). The transcripts of *TaSRS1-1D* and *TaSRS4-7D* were highly accumulated at 6 h of drought, and *TaSRS5-7D* showed transient up-regulation at the early stage of heat treatment (1 h), whereas *TaSRS2-3D* was up-regulated across the treatments. These patterns were further validated by qRT-PCR, which showed significant upregulation of TaSRS1-1D and TaSRS2-3D at 6–12 h of drought, while TaSRS3-5B and TaSRS4-7A showed progressive downregulation with increasing stress duration, and TaSRS5-7A showed an early peak followed by a decline (Figure 9B–F). These results suggest the temporal differentiation of roles in stress perception and regulation. The *TaSRS1-1D* and *TaSRS2-3D* belong to the LRP1-like lineage, while the repressed or temporally expressed genes, i.e., *TaSRS3-5B*, *TaSRS4-7A*, and *TaSRS5-7A*, were grouped in the SHI/STY-like branch. These results suggest that phylogeny could be linked to functional divergence, with one clade predominantly participating in stress responses and the other in growth and development in plants [12,21]. Similar differences can be observed in their promoter composition; TaSRS1-1D and TaSRS2-3D possess a high density of ABRE and other stress-responsive CREs, with binding sites for NAC ERF, WRKY, and HSF transcription factors, which are known for their roles in ABA- and ROS-mediated drought signalling. *TaSRS3-5B* and *TaSRS4-7A* promoters were enriched for auxin- and development-related CREs, with few canonical stress TFBSs, consistent with their suppression, as growth-promoting pathways are attenuated during drought. Taken together, analogous lineage-based responses reported for *SRS* genes in plants suggest the LRP1-like *TaSRS* genes, composed of stress-responsive regulatory elements, act as positive regulators of drought adaptation, while SHI/STY-like members remain primarily developmental regulators as their expression is actively suppressed during drought [11,21,23,29,55,69].

#### 2.8.3. Salt-Responsive Expression

**Figure 10:**
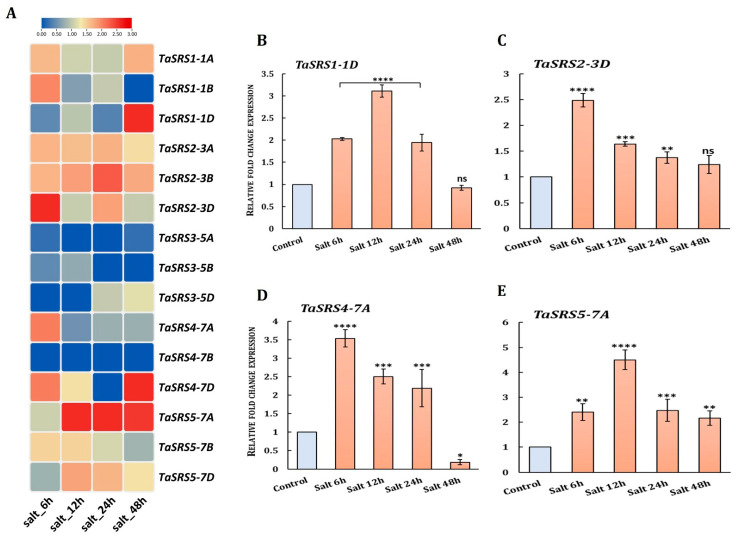
**Expression analyses of *TaSRS* genes during salinity stress conditions.** (**A**) The figure shows the temporal fold change expression profiles (FPKM) of *TaSRS* genes during salinity stress, calculated against the control. The high level of expression indicated by red and blue colours indicates lower expression. The heatmap was generated using TBtools. (**B**–**E**) The bar graphs show transcript accumulation of selected *TaSRS* genes under salinity stress at 1 and 6 h of the 150 mM NaCl treatment. qRT-PCR was performed using total RNA of root tissues. *TaARF* was used as an internal control. The experiment was conducted in triplicate, and the data were represented as mean ± SD. Asterisks denote the level of significance compared with control (* *p* ≤ 0.05; ** *p* ≤ 0.01; *** *p* ≤ 0.001; **** *p* ≤ 0.0001; ns, not significant), determined by one-way ANOVA (Fisher’s and Dunnett’s post-test).

Salinity induced a partly overlapping but distinct subset of *TaSRS* genes. Transcripts of *TaSRS1-1D*, *TaSRS2-3D*, *TaSRS4-7A* and *TaSRS5-7A* were highly accumulated, whereas *TaSRS2-3A*, *TaSRS2-3B* and HGs of *TaSRS3* were less responsive or slightly down-regulated (Figure 10A). The qRT-PCR analysis validated these results (Figure 10B–E). *TaSRS1-1D* and *TaSRS2-3D* (LRP1-like) showed approximately a two- to threefold increase in expression at 6–12 h, and *TaSRS4-7A* and *TaSRS5-7A* were strongly up-regulated (four- to five-fold), which persisted into later time points before down-regulation by 48 h. These results indicate that these genes function during the early and intermediate phases of salt stress. From a phylogenetic perspective, the presence of both LRP1-like and SHI/STY-like members among the salt-inducible genes indicate that salt-responsive functions arose independently within both lineages, consistent with cassava, soybean, sesame and others, where only specific SRS paralogs respond robustly to salt stress [23,56,69,70].

## 3. Materials and Methods

### 3.1. Identification of TaSRS Genes

We identified *TaSRS* genes in the bread wheat genome using a combination of homology-based and domain-specific strategies. Initially, the SRS protein sequences from rice and Arabidopsis were retrieved from the Ensembl Plants database (IRGSP-1) and The Arabidopsis Information Resource (TAIR) [71,72], respectively, and used as queries in the BLASTp search against the reference wheat genome (IWGSC RefSeq v2.1) in the Ensembl Plants database (release 56). Identified sequences were screened for the occurrence of DUF702/RING-like zinc-finger motif using the InterPro, Pfam and SMART databases [73]. Protein sequences that possessed RING-like zinc-finger domains were considered as TaSRS proteins and used for downstream analysis.

### 3.2. In Silico TaSRS Protein Characterization

Various protein features such as peptide length (PL), molecular weight (MW), grand average of hydropathicity (GRAVY), and theoretical isoelectric point (pI) were calculated using the Expasy-ProtParam tool [74]. Subcellular localization was predicted by the DeepLoc 2.1 web-tool and cross-validated using the WoLF PSORT (https://wolfpsort.hgc.jp/, accessed on 20 September 2025). The presence of signal peptides and nuclear localization signal was assessed using the SignalP 6.0, and transmembrane domains were assessed using the TMHMM 2.0, respectively.

### 3.3. Chromosomal Location, Nomenclature, and Gene Structure Analyses

The chromosomal and sub-genomic coordinates of *TaSRS* genes were retrieved from the *T. aestivum* reference genome (IWGSC RefSeq v2.1) via the Ensembl Plants database. Physical mapping visualization on chromosomes was generated using the MG2C_v2.1. Gene nomenclature for *TaSRS* followed the standardized guidelines of the International Wheat Genome Nomenclature Committee (98 Cat_Revised), ensuring consistent, universally recognized gene symbol assignments that reflect their chromosomal locations [30].

For gene structure characterization, the genomic and their corresponding coding sequences (CDSs) were downloaded from Ensembl Plants [71]. The exon-intron organization, including the distribution and length of untranslated regions (UTRs) and intron phases, were visualized using the Gene Structure Display Server 2.0.

### 3.4. Motifs and Conserved Domains Analyses

Conserved motifs were identified in the TaSRS protein using the MEME Suite (https://meme-suite.org/meme/, accessed on 22 September 2025) with default settings, except for limiting the maximum number of motifs to 10. The significance of identified motifs was assessed based on E-values and positional conservation across the sequences.

The SMART tool (https://smart.embl.de/help/smart_about.shtml, accessed on 25 September 2025) and the NCBI Conserved Domain Search (CD-Search) were employed to annotate conserved domains in the TaSRS proteins. The Tbtools-II software (v2.423) was used to visualize the domain organization [75].

### 3.5. Sequence Alignment, Phylogeny, and Synteny Analyses

Full-length TaSRS protein sequences were aligned using the Multalin web tool (http://multalin.toulouse.inra.fr/multalin/, accessed on 10 October 2025). Conserved residues, functional domains, and motifs were identified through visual inspection and manually annotated.

To explore shared evolutionary ancestry and genomic collinearity, we performed synteny analyses with the Circoletto web tool (Circoletto @ the BAT cave) (https://bat.infspire.org/circoletto/, accessed on 11 October 2025). Full-length SRS protein sequences from *Aegilops tauschii*, *Triticum urartu*, *T. aestivum*, *O. sativa*, *Z. mays* and *A. thaliana* were used as inputs into the Circoletto at default parameters with a minimum alignment score threshold of 180 bits.

The phylogenetic relationships among full-length SRS proteins from *Ae. tauschii*, *T. urartu*, *T. aestivum*, *O. sativa*, *Z. mays* and *A. thaliana* were inferred using multiple sequence alignment (MSA) generated by the MUSCLE algorithm incorporated within the MEGA 11 software. The neighbour-joining phylogenetic tree was constructed and subsequently visualized using the Interactive Tree of Life (iTOL) platform (https://itol.embl.de/upload.cgi, accessed on 12 October 2025).

### 3.6. Identification of CREs and TFBSs Analyses

Promoter regions extending 2000 base pairs upstream from the translation start site for each *TaSRS* gene were retrieved from the Ensembl Plants database. These sequences were subjected to in silico screening for CREs using the PlantCARE web tool (https://bioinformatics.psb.ugent.be/webtools/plantcare/html/, accessed on 15 October 2025). Detected CREs were systematically classified by their known or predicted biological functions, including responses to stress, hormonal signalling pathways, and developmental regulation. To further elucidate regulatory networks, potential TFBSs within these promoters were predicted using the PlantRegMap web tool (https://plantregmap.gao-lab.org/binding_site_prediction.php, accessed on 17 October 2025). The Tbtools-II software (v2.432) was used to visualize the distribution landscape of predicted CREs and TFBSs [75].

### 3.7. Interaction Analyses

To investigate post-transcriptional regulation, miRNA interactions were predicted for both *TaSRS* coding sequences and upstream promoter regions. These sequences were analyzed using the psRNATarget server (https://www.zhaolab.org/psRNATarget/, accessed on 18 October 2025) with default parameters including known miRNAs from *T. aestivum*. The Tbtools-II software (v2.432) was used to visualize the predicted miRNA-target interactions [75].

Protein–protein interaction (PPI) networks of TaSRS proteins were predicted using the STRING v12.0 server with default parameters. A full STRING network type was applied, and interactions were considered significant at a medium confidence threshold (score > 0.4). The resulting PPI networks were visualized and analyzed using the Cytoscape 3.10.4 software.

### 3.8. Gene Expression Analysis

Next-generation RNA-seq datasets of allohexaploid bread wheat were obtained from the Unité de Recherche en Génomique-Info (URGI) repository. Expression profiling of *TaSRS* genes was conducted across developmental stages in diverse tissues, including grain, spike, stem, leaf, and root, with two biological replicates. Additional RNA-seq data from Liu et al. (2015) [76] were used to assess *TaSRS* gene responses in leaf tissues under heat, drought, and combined heat-drought stress at 1 h and 6 h time points. Salinity stress expression patterns were analyzed using root transcriptomes from Zhang et al. (2016) [77] after treatment with 150 mM NaCl at different time intervals (6, 12, 24, 48 h). All datasets were processed through the Trinity pipeline, and gene expression was quantified in fragments per kilobase per million mapped reads (FPKM) [78]. Genes were identified as differentially expressed using a twofold change threshold and a FDR cutoff of 0.05. Expression heatmaps were generated using the TBtools software [75].

### 3.9. Plant Materials and qRT-PCR Analysis

To validate the RNA-seq expression profiles, qRT-PCR was performed for selected *TaSRS* genes using protocols established in our laboratory [79]. Seeds of *T. aestivum* were grown hydroponically after surface sterilization with 0.1% HgCL_2_ and subjected to drought (20% polyethylene glycol) and salinity (150 mM NaCl) treatments. Shoot and root tissues were used for total RNA isolation and cDNA synthesis for gene expression analysis under drought and salinity stress treatments, as described previously [79,80]. qRT-PCR reactions were performed using gene-specific primers and TB Green^®^ Premix Ex Taq™ II (Tli RNase H Plus) (Takara Bio Inc., Shiga, Japan) on the CFX96 Real-Time PCR system (Bio-Rad Laboratories Inc., Hercules, CA, USA). Relative fold-change expression levels were calculated using the 2^−ΔΔCT^ method, where *TaARF* was used as an internal reference gene [39,79]. All assays were conducted in three biological replicates, and one-way ANOVA was used to determine the statistical significance.

## 4. Conclusions

The study delineates the composition, structural features, and evolutionary relationships of the *TaSRS* genes in bread wheat. These properties are also linked to distinctive expression signatures under salinity and drought stress conditions. Integrating promoter architecture, phylogenetic placement, transcriptomic, and qRT-PCR data highlighted that a limited subset of TaSRS members consistently responds to drought and salinity stress, suggesting lineage- and context-specific regulatory roles in stress adaptation. However, the precise role of each *TaSRS* gene needs to be validated using overexpression or knock-out approaches in future studies.

## Figures and Tables

**Table 1:** Physicochemical properties of TaSRSs of bread wheat.

Gene Name	Chromosome	CDS Length	Exons	Protein Length	MW (kDa)	pI	Sub-Cellular Localization
*TaSRS1-1A*	1A	0945	3	314	32.54	8.89	Nucleus
*TaSRS1-1B*	1B	0942	3	313	32.58	8.89	Nucleus
*TaSRS1-1D*	1D	0942	3	313	32.44	8.89	Nucleus
*TaSRS2-3A*	3A	0744	2	247	25.09	8.43	Nucleus
*TaSRS2-3B*	3B	1059	2	352	35.48	7.72	Nucleus
*TaSRS2-3D*	3D	1047	2	348	35.26	7.70	Nucleus
*TaSRS3-5A*	5A	0993	2	330	33.92	8.96	Nucleus
*TaSRS3-5B*	5B	0993	2	330	33.92	8.96	Nucleus
*TaSRS3-5D*	5D	0993	2	330	33.92	8.96	Nucleus
*TaSRS4-7A*	7A	0948	2	315	32.50	8.16	Nucleus
*TaSRS4-7B*	7B	1008	3	335	34.64	7.71	Nucleus
*TaSRS4-7D*	7D	948	2	315	32.39	8.16	Nucleus
*TaSRS5-7A*	7A	1041	2	346	34.38	8.68	Nucleus
*TaSRS5-7B*	7B	1041	2	346	34.47	8.41	Nucleus
*TaSRS5-7D*	7D	1041	2	346	34.43	8.68	Nucleus

## Data Availability

The original contributions presented in this study are included in the article and Supplementary Materials. Further inquiries can be directed to the corresponding authors.

## Supplementary Materials

The following supporting information can be downloaded at: https://www.mdpi.com/article/10.3390/ijms27073269/s1.
