# Supplementary figures and images for: SHORT INTERNODE (SHI)-Related Sequence Genes in Bread Wheat: Molecular Characterization and Expression Analyses Suggest Their Role in Abiotic Stress Response
Source: Int J Mol Sci. 2026 Apr 3;27(7):3269. doi: 10.3390/ijms27073269 (PMC13073955; doi:10.3390/ijms27073269)

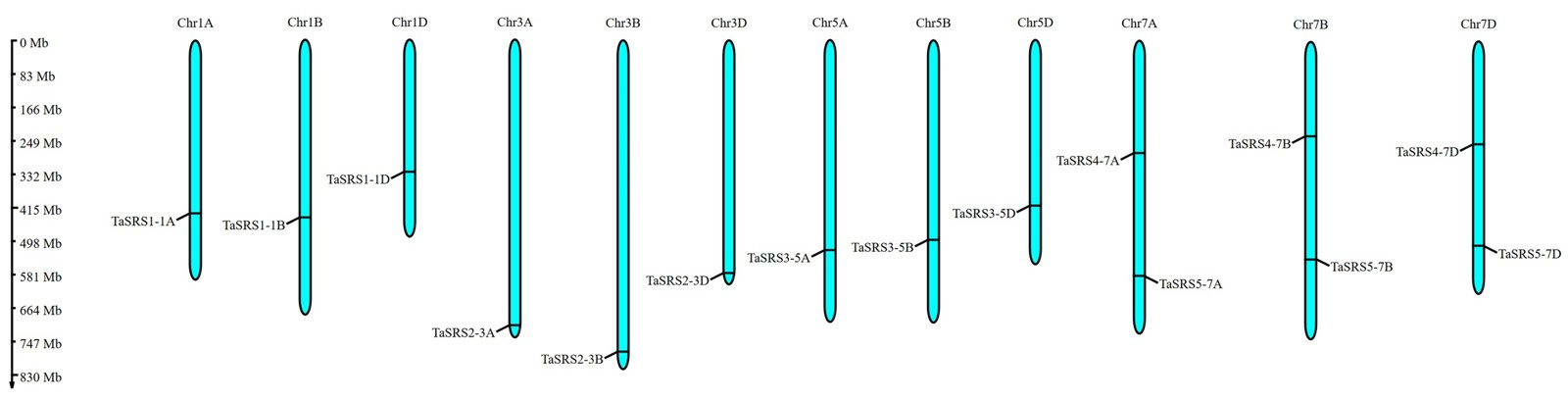

Supplement: Supplementary file 1 [file ijms-27-03269-s001.zip › Sup files/Figure S1.jpg]
